# Evaluation of long-term consequences among snakebite survivors in rural Kenya including validation of a functional limitations assessment tool

**DOI:** 10.1371/journal.pntd.0014259

**Published:** 2026-04-28

**Authors:** Cecilia Ngari, Stian Cattell-Ravdal, Steven A. Wasonga, Rafael Cuginotti de Oliviera, Tonny O. Ngage, Luka Dolinaj, Loreta A. Asila, Jonathan Steinhorst, Peter G. Mwethera, Lynn Kitwan, John H. Amuasi, David G. Lalloo, Leslie M. Aglanu, George O. Oluoch, Ymkje Stienstra

**Affiliations:** 1 Kenya Snakebite Research and Intervention Centre, Kenya Institute of Primate Research, Ministry of Health, Nairobi, Kenya; 2 University of Groningen, Department of Internal Medicine/Infectious Diseases, University Medical Centre Groningen, Groningen, The Netherlands; 3 Centre for Snakebite Research and Interventions, Liverpool School of Tropical Medicine, Liverpool, United Kingdom; 4 County Ministry of Health and Sanitation, Kitui, Kenya; 5 Department of Implementation Research, Bernhard Nocht Institute for Tropical Medicine, Hamburg, Germany; 6 Global Health and Infectious Diseases Research Group, Kumasi Centre for Collaborative Research in Tropical Medicine, Kumasi, Ghana; 7 Department of Global Health, School of Public Health, Kwame Nkrumah University of Science and Technology, Kumasi, Ghana; 8 Division for Tropical Medicine, Department of Medicine, University Medical Centre Hamburg-Eppendorf, Hamburg, Germany; Fundação de Medicina Tropical Doutor Heitor Vieira Dourado: Fundacao de Medicina Tropical Doutor Heitor Vieira Dourado, BRAZIL

## Abstract

**Background:**

Snakebite envenoming, a neglected tropical disease, causes functional limitations and disability, yet the burden and optimal assessment methods remain unclear. We describe the frequency and severity of disability and functional limitation among snakebite survivors in Kenya and validate a tool to evaluate interventions and guide rehabilitation.

**Methods:**

Snakebite survivors whose incident occurred at least six months prior to the day of survey and healthy community controls in Kitui County, Kenya, were sampled and assessed using the World Health Organization Disability Assessment Schedule (WHODAS 2.0) and the Buruli Ulcer Functional Limitation Scale (BUFLS). The BUFLS was tested for construct validity based on five a priori hypotheses and discriminant validity.

**Results:**

In total, 140 snakebite survivors and 57 community controls were included. Of the 140 survivors, 87 (62.1%) were categorised as having more severe envenoming (SBE) based on reporting multiple clinical symptoms, while 53 (37.9%) were categorised as snakebite only (SB) having reported localised redness or no symptoms at all. Among survivors with more severe envenoming, 49% reported mild disabilities, compared to 32% in the snakebite survivors who presented with less severe symptoms of envenoming. The highest levels of disabilities were observed in the domains of mobility, participation, and life activities. In the control group, 12% reported mild disabilities, reflecting real-world background disability common in rural sub-Saharan African communities. The BUFLS met the predefined construct validity criteria and displayed good discrimination between snakebite survivors and controls. The activities mainly affected were those requiring gross motor skills within the BUFLS domains of food preparation, personal care, work and mobility.

**Conclusion:**

Snakebite survivors exhibit high rates of long-term disability and gross motor skill functional limitations, which must be considered when designing and evaluating public health interventions improving management and rehabilitation. The BUFLS provides a valid tool for assessing functional limitations in regions where cytotoxic envenoming predominates.

## Author summary

Snakebite is a serious neglected tropical disease that often leaves survivors with long-term disabilities, but this burden is poorly measured. Using a standardized WHO (World Health Organization) tool to measure health and disability in rural Kenya, we found that over three-quarters of snakebite survivors reported difficulties with daily activities, compared to just over a quarter of people who had not been bitten. We also tested and validated a new practical tool, originally designed for a similar disabling condition, to assess specific functional limitations like fetching water or farming. We validated the tool for future use in our setting, where snakebites often cause local tissue damage. Our findings showed that snakebite is associated with a significant burden of long-term disability, showing that comprehensive care must extend beyond acute antivenom treatment to include scalable rehabilitation services. The validated assessment tool also provides a practical method to guide the activities in rehabilitation programmes and quantitatively measure the effectiveness of their interventions.

## Introduction

Snakebite envenoming (SBE) is a critical but neglected tropical disease, disproportionately affecting rural communities in tropical and subtropical regions [1]. It is estimated that SBE causes between 81,000–138,000 deaths globally each year, while over 400,000 survivors endure permanent disabilities including limb amputations and blindness [1–3]. However, the absence of routine clinical follow-up and limited research attention have resulted in poor understanding of the long-term outcomes of snakebite envenoming [4–6]. Some effects, including psychological complications, may also appear long after the initial incident [7].

Sub-Saharan Africa (SSA) bears a disproportionate burden of SBE-related morbidity and mortality [6,8] due to a confluence of factors: the high prevalence of medically significant snake species; increased human-snake interaction through agricultural and pastoralist activities, and poor housing conditions; limited access to health services; and frequent antivenom shortages [2,7,9].

Not all snakebites result in envenoming; up to 50% of bites from venomous species may be “dry” or with insufficient venom injected to cause clinical effects [10,11]. When envenoming does occur, clinical presentations can vary depending on the venom type which is broadly classified as cytotoxic, haemotoxic and neurotoxic [12,13]. Haemotoxicity and neurotoxicity typically cause acute complications, whereas cytotoxicity tends to lead to long term morbidity due to contractures and amputations [12,14]. Nevertheless, all snakebites, even dry bites, may lead to psychological sequelae consisting of post-traumatic stress disorder, anxiety or depression, and healthcare costs that perpetuate socioeconomic hardship [15,16].

A community-based study in Ghana found an increased prevalence of mainly mild to moderate disabilities amongst snakebite survivors. Common issues included joint dysfunction and scarring, limiting mobility, daily functioning and social participation [17]. In Kenya, an estimated 15,000 people are envenomed annually resulting in approximately 800 deaths each year [18]. Medically important species in the country include cobras (*Naja* spp.), mambas (*Dendroaspis* spp.), puff adders (*Bitis arietans*) and burrowing asps (*Atractaspis* spp.).

We looked at the prevalence and severity of disabilities and functional limitations amongst community members with and without a history of snakebite in Kitui County, Kenya. We used the World Health Organization Disability Assessment Schedule to assess levels of disability and examined the validity of the Buruli Ulcer Functional Limitation Scale to clarify the nature of functional limitations driving these disabilities. In addition, findings from this region in Kenya were compared with data obtained from Ghana where similar methodology was used to assess the influence of predominantly cytotoxic envenoming in the study regions on the pattern of disability.

## Methodology

### Ethics statement

Ethics approval was obtained from the Moi Teaching and Referral Hospital/Moi University College of Health Sciences Institutional Research and Ethics Committee (MTRH/MU-IREC) approval number FAN: 0004853, the National Commission for Science, Technology and Innovation (NACOSTI); license number NACOSTI/P/24/414351, the Kitui County Ministry of Health and Sanitation; REF CGKTI/MOH/ADM/8/3(232), and the University Medical Centre Groningen (METc 2024/241, waiver). Adults provided written or thumb printed consent after being informed about the study, while adolescents aged 13–17 gave written assent alongside a written guardian consent. Community Health Practitioners (CHPs) acted as impartial witnesses during the consent process.

### Study setting and population

A population-based cross-sectional study was conducted in Kitui County, the sixth largest of Kenya’s 47 counties in the months of April and May 2024. Kitui was identified as a high-incidence region for snakebite envenoming based on retrospective analyses of Kenya Health Information System (KHIS) data from 2020 to 2024. Located in the eastern region, the county spans a land area of 30,496 square kilometres. Kitui has an estimated population of 1,136,187, with agriculture serving as the primary source of livelihood [19,20].

The study population consisted of residents of Kitui County including individuals with a history of snakebite envenoming occurring between six months and five years prior to enrolment and community controls with no prior history of snakebite. Participants aged 13–17 years were interviewed in the presence of a parent or guardian in accordance with ethical requirements. Community Health Units (CHUs) with the highest reported cases based on KHIS were selected for participant recruitment at the community level. Survivors were identified through a multi-stage case-finding strategy. High-burden wards were first selected in each sub county, after which their respective public health officers linked the study team to community health promoters who compiled a complete list of known snakebite survivors within their community units through community informed case finding. Community health promoters (CHPs) were asked to record all identified snakebite survivors, irrespective of presence or absence of long-term sequelae. Individuals were convened at predetermined sites, where their snakebite history was confirmed by the study team through clinical documentation or participant interviews prior to enrolment. Participants under 13 years of age and those unable to accurately recall and describe their snakebite experiences were excluded from the study.

Following the enrolment of snakebite survivors, controls were then recruited from the same communities as survivors, matched for community of residence, 5-year age range, and sex. CHPs identified eligible controls from household listings within the same community units. The data collection team proceeded community by community, enrolling all eligible individuals at each site before moving to the next.

We aimed to include at least 129 snakebite survivors and 43 community controls to provide ≥80% power to detect a four-fold difference in the prevalence of disability between these two groups. This sample size was calculated assuming an alpha value of 0.05 and an estimated functional limitation and disability prevalence of 4.6% in Kitui County based on the 2019 Kenya Population and Housing Census and adjusted for the 3:1 ratio of snakebite survivors to community controls. A ‘snakebite survivor’ was defined as any person who had been bitten by a snake or had snake venom spat on into the eye. Visual aids depicting common snake species (including both venomous and non-venomous snakes) in the region were used to assist survivors in identifying the snake involved.

### Data collection

A structured survey was developed, pre-tested, and administered in the local vernacular languages (Swahili and Kamba). The survey, based on Aglanu et al, consisted of sociodemographic information, snakebite history, medical care received and functional and psychosocial assessments [17].To evaluate the functional and psychosocial status of both snakebite survivors and community controls, we employed two standardized tools, the World Health Organization Disability Assessment Schedule (WHODAS 2.0) and the Buruli Ulcer Functional Limitation Scale (BUFLS).

We used the 36-item version of the WHODAS 2.0 that encompasses six domains: cognition, mobility, self-care, getting along with others, life activities, and participation in society. Participants rated the difficulty of completing different tasks in the 30-day period preceding the date of the assessment on a 5-point Likert scale ranging from no difficulty (0 points) to extreme difficulty (4 points). For participants who neither worked nor studied, total scores were calculated using 32 items, after excluding the items pertaining work and school. The SPSS syntax for automatic computation provided in the WHO manual was used to calculate item scores within each domain. The total score across all six domains was then converted into a metric ranging from 0 to 100, where 0 denotes no disability and 100 indicates full disability [21]. Accordingly, WHODAS 2.0 overall scores were categorized according to the International Classification of Functioning Disability and Health (ICF) classification as follows: no problem (0–4%), mild problem (>4–24%), moderate problem (>24–49%), severe problem (>49–95%), and extreme problem (>95%) [22].

Previous studies have successfully utilized WHODAS 2.0 in similar geographical and disease settings, confirming its suitability and adaptability. Additionally, the WHODAS 2.0 scores align with the International Classification of Functioning, Disability and Health (ICF) model, an extensive and universally accepted classification system that enables clinicians and field workers to systematically describe and categorize patients’ functioning and disability [23–27].

The Buruli Ulcer Functional Limitation Score (BUFLS) is a clinical assessment tool originally designed to measure functional limitations resulting from Buruli ulcer disease. In this study, the BUFLS was used as a model to assess functional, physical, and psychosocial limitations following snakebite envenomation (SBE). This was justified because both Buruli ulcer and snakebite envenomation share similar clinical complications, including tissue necrosis, potential amputations, contractures, chronic pain, stigma and psychosocial distress.

The original BUFLS has been effectively administered to diverse patient cohorts in Ghana and Benin, demonstrating high reliability, consistency, and validity for assessing functional limitations. It exhibits excellent inter-observer reliability (ICC = 0.86) and internal consistency (Cronbach’s alpha = 0.90), ensuring stable and dependable measurements across different evaluators and contexts. Its validity is well-supported by strong correlations with global impressions of functional limitations, range of motion restrictions, and social impacts [28].

Each of the 19 items in the BUFLS was scored as “easily” (0 points), “with difficulties” (1 points), or “not possible” (2 points), with non-applicable items excluded. Items rated ‘not applicable’ referred to activities the participant does not perform for reasons unrelated to their condition. The individual score was calculated as the percentage of applicable activities rated as “with difficulties” or “not possible.” The total score was expressed as a percentage ranging from 0 to 100, with 0 indicating no functional limitations. The individual functional limitation score was not calculated for participants with more than six items rated as not applicable.

### Data analysis

All responses were electronically captured using REDCap software on password-protected devices and analysis performed using IBM SPSS Statistics version 25 and R version 4.4. Descriptive Statistics was used to summarize participant characteristics, snakebite specifics, and assessment scores.

The snakebite survivors’ group was categorized into two groups by severity of the initial symptoms. Participants who reported multiple clinical symptoms (redness, swelling around the bite site, haematoma, bleeding for more than an hour or other systemic signs) were categorised as Snakebite Envenoming -SBE. Those who reported only localised redness around the bite site or no symptoms at all were categorized as Snakebite -SB.

To test the validity of the BUFLS as a tool for measuring functional limitations in our study population, the following a priori hypotheses were used to test for construct validity.

i. The BUFLS shows a strong positive correlation with WHODAS total disability scores within the expected range (0.4 ≤ ρ ≤ 0.8).
ii. The BUFLS is further supported by a positive correlation with the WHODAS domain on mobility (0.4 ≤ ρ ≤ 0.8).
iii. BUFLS scores are significantly higher among individuals who experienced more severe signs of envenoming (SBE versus SB group)
iv. BUFLS scores are significantly higher in participants with a visible disability as assessed by the researcher.
v. Higher BUFLS scores are associated with self-reported disruption in work or school participation.

The tool was considered to demonstrate adequate construct validity if the results supported at least four out of the five hypotheses. To assess the discriminant validity of the BUFLS tool, we tested whether functional limitation scores differed between previous snakebite patients and healthy controls. Floor and ceiling effects were also tested and considered significant if >15% of participants scored lowest or the highest possible score respectively. Prevalence ratios (PR) were estimated as simple ratios of proportions using the epi.2by2() function in the epiR package in R.

## Results

### Study population

A total of 212 snakebite survivors were initially identified, of whom 140 (47.1% male) met the eligibility criteria and were enrolled, alongside 57 community controls (43.9% male).

**Fig 1:**
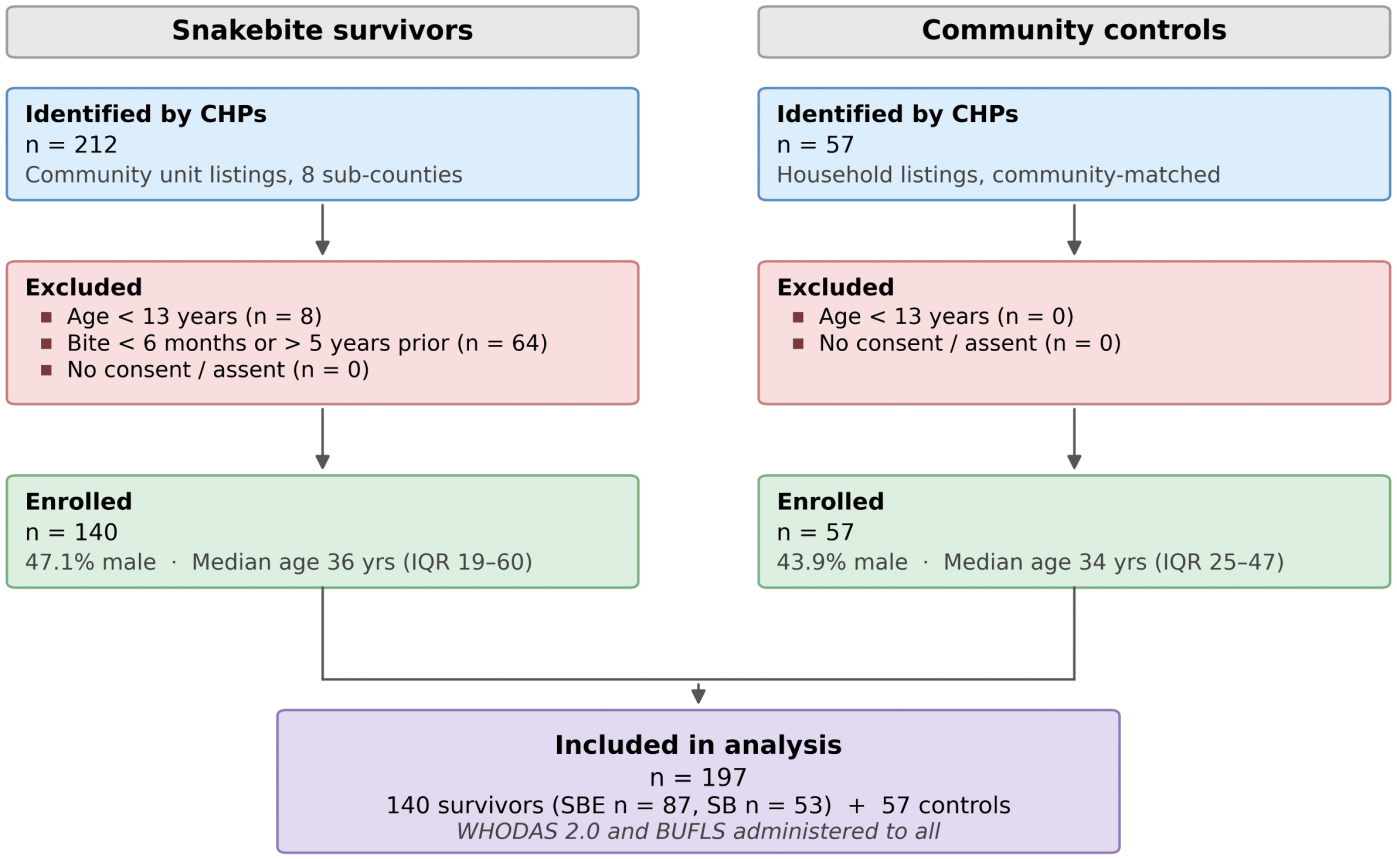
Participant recruitment and enrolment flow diagram.

The flow of participants through identification, screening, and enrolment is shown in Fig 1 below.

Data collection took place between April and May 2024 with elaborated demographic characteristics and visible sequelae of the study population presented in Table 1. The median age of survivors was 36 years (IQR:19–60) and the median (IQR) time since the snakebite was 3.5 (1.6-4.5) years. Only 11 (7.9%) had experienced two or more bites. A total of 87 survivors (62.1%) were categorised as SBE, reporting multiple clinical symptoms, whereas 53 (37.9%) were categorised as SB with either no symptoms or only mild local redness. Based on the accounts of the snake spotted during the incident and the visual reference materials provided, the red spitting cobra (*N. pallida*) was most frequently identified (68 cases, 60.2%), followed by the puff adder (*B. arietans*) (22 cases, 19.5%).

**Table 1:** Demographic characteristics and visible sequelae of study population (N = 197).

Demographic characteristics		Snakebite survivors n (%)	Controls n (%)
		140 (71.1)	57 (28.9)
Subcounty	Mwingi Central	34 (24.3)	12 (21.1)
	Kitui South	31 (22.1)	15 (26.3)
	Kitui Central	30 (21.4)	8 (14.0)
	Mwingi North	23 (16.4)	3 (5.3)
	Kitui East	10 (7.1)	7 (12.2)
	Kitui West	5 (3.6)	5 (8.8)
	Mwingi West	4 (2.8)	4 (7.0)
	Kitui Rural	3 (2.1)	3 (5.1)
Gender	Male	66 (47.1)	25 (43.9)
	Female	74 (52.9)	32 (56.1)
Age (years)	Median (IQR)	36 (19 – 60)	34 (25 – 47)
Main Work Status	Employed	17 (12.1)	17 (29.8)
	Self-employed	69 (49.3)	32 (56.1)
	Student	36 (25.7)	5 (8.8)
	Retired	1 (0.7)	1 (1.8)
	Unemployed (health)	8 (5.7)	0 (0.0)
	Unemployed (other)	5 (2.1)	0 (0.0)
	Other work status*	4 (2.9)	2 (3.5)
Visible sequelae (any cause)	No	92 (65.7)	56 (98.2)
	Yes	48 (34.3)	1 (1.8)
Type of visible sequelae	Amputation	9 (6.4)	0 (0.0)
	Scar tissue without ROM **impairment	15 (10.7)	0 (0.0)
	Scar tissue with ROM impairment	14 (10.0)	0 (0.0)
	Eye damage	1 (0.7)	0 (0.0)
	Paresis	0 (0.0)	1 (1.8)
	Contracture	8 (5.7)	0 (0.0)
	Other physical sequela	1 (0.7)	0 (0.0)

*Temporary work or paid tasks.

**Range of Motion

### Disability among snakebite survivors

A total of 48 (34.2%) snakebite survivors had visible sequelae as reported by the researchers with scar tissue being most common. About 48% of those with scar tissue had an associated range of motion impairment.

Disabilities were more commonly reported by participants than would have been expected from the observed physical sequelae by the researchers. Snakebite survivors demonstrated significantly higher prevalence of disabilities compared to controls as shown by WHODAS 2.0 scores (77.1% vs. 28.1%; PR, 2.75; 95% CI, 1.80 - 4.20).

Participants in the SBE group recorded a higher prevalence of disabilities when compared to those in the SB group (86.2% vs. 62.3%; PR 1.38; 95% CI, 1.1–1.74) with the SB group being over twice as likely to report disability (WHODAS 2.0 > 0) compared to controls (PR = 2.22, 95% CI: 1.39–3.53), and the SBE group, reporting more than three times the prevalence (PR = 3.07, 95% CI: 2.01–4.69).

**Fig 2:**
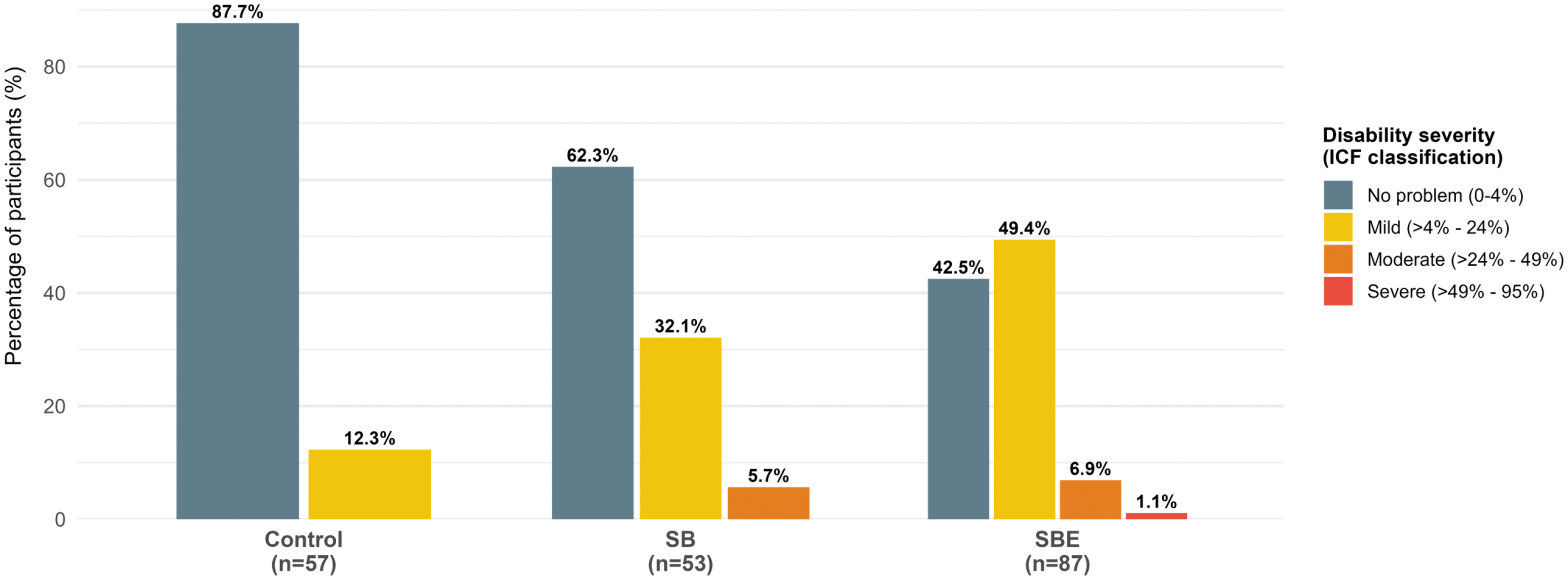
Prevalence of disability severity among the three study groups (Controls, n = 57, SB, n = 53, and SBE, n = 87) as measured by the WHODAS 2.0. Snakebite survivors in the SBE group had a more severe envenoming than survivors in the SB group based on history.

Based on the WHODAS 2.0 scores, mild and moderate disabilities were more prevalent among snakebite survivors compared to controls. Moderate to severe disability was observed in 8% of SBE participants, compared to 5.7% in SB participants (Fig 2).

**Fig 3:**
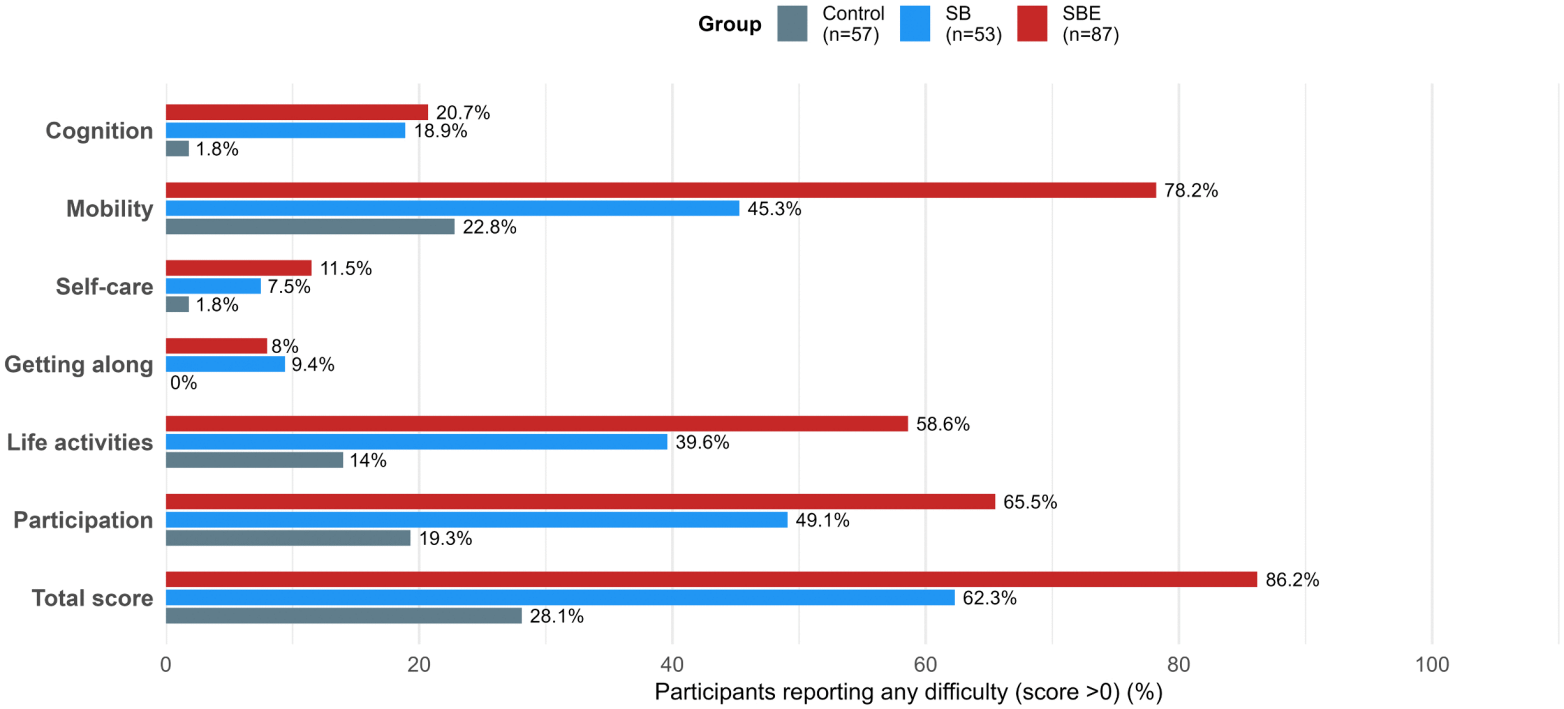
WHODAS 2.0 domain scores among the three study groups (Controls, n = 57; SB, n = 53; SBE, n = 87). The x-axis represents the percentage of participants reporting any degree of difficulty (>0 score) within each WHODAS 2.0 domain.

Within the WHODAS 2.0 domains, the SBE group recorded higher scores across all categories compared to the SB and control groups. The highest levels of disabilities were observed in the domains of participation, mobility, and life activities. Fewer limitations were seen in cognition, getting along, and the self-care domains (Fig 3).

### Validity of the BUFLS

Four of the five *a priori* hypotheses that were formulated to assess the construct validity of the BUFLS in measuring functional limitations after a snakebite were strictly supported, thus meeting the predefined criterion for construct validity which required at least four of five.

Hypothesis 1: A strong positive correlation of 0.76 (Spearman, n = 196, p < 0.001) was observed between individual WHODAS 2.0 scores and BUFLS scores, suggesting that higher levels of disability on the WHODAS 2.0 were correlated with greater functional limitation as measured by BUFLS.

Hypothesis 2: BUFLS showed a very strong positive correlation (Spearman’s ρ 0.87) with the WHODAS mobility domain. This exceeded the predefined upper bound for construct validity (0.4 ≤ ρ ≤ 0.8) and is therefore not counted as strictly supported under the pre-specified criterion.

Hypothesis 3: The proportion of individuals with BUFLS > 0 (indicating the presence of functional limitation) was higher in the SBE group (76/86, 88.4%) compared to the SB group (39/53, 73.6%). The median BUFLS scores were higher in the SBE group (13.2; IQR: 5.3–24.7) compared to the SB group (11.1; IQR: 0–29.4, p < 0.001).

Hypothesis 4: The median (IQR) BUFLS score was 26.5 (10.5-36.7) in those with visible sequelae (n = 49), compared to 5.3 (0-14.7) in participants without visible sequelae (n = 147). Prevalence ratio was 1.42 (95%CI 1.22-1.66) when those with visible sequelae (44/49) were compared with those without (93/147).

Hypothesis 5: A total of 30 participants reported disruption to their daily activities following snakebite: 27 reported changes in work activities, 2 reported delays or interruptions in school attendance and 1 reported experiencing impacts on both school and work responsibilities. Participants who experienced these changes had substantially higher BUFLS scores (median 28.5, IQR: 15-36.8) compared to those without (median 5.26, IQR: 0-15.8, MWU p < 0.001).

In testing for discriminant validity, the BUFLS scores were significantly higher in snakebite survivors compared to community controls (Median (IQR) 0 (0-5.3) in controls vs 13.2(3.0-16.8) in snakebite survivors (MWU, p < 0.001).

### Functional limitations identified by the BUFLS

Functional limitations, as measured by the BUFLS were most prevalent in the mobility domain, where more than half of survivors reported difficulty running (55.7%, n = 78) and walking uphill (50.7%, n = 71), compared to 19.3% (n = 11) of controls for both activities. Personal care and food preparation activities were less frequently affected. Snakebite survivors consistently reported higher rates of limitation than controls across all activities. The number of participants who rated individual BUFLS activities as ‘not applicable’, indicating that the activity was not performed for reasons unrelated to their condition, is reported for each activity in S1 Table. Only one participant had more than six items rated as not applicable and an individual BUFLS score was therefore not computed for this participant.

Table 2 below presents the proportion of snakebite survivors and controls reporting residual functional limitations across all 19 BUFLS activities, grouped by type of activity.

**Table 2:** Distribution of residual functional limitations among snakebite survivors and controls across BUFLS activities.

			Snakebite Survivors (n = 140) Median BUFLS (IQR) 13.2(3.0-16.8)		Controls (n = 57) Median BUFLS (IQR) 0 (0-5.3)		
Type of activity	Activity	Extremity	No Difficulties (BUFLS = 0)	Residual Functional Limitations (BUFLS>0)	No Difficulties (BUFLS = 0)	Residual Functional Limitations (BUFLS>0)	Prevalence Ratio (95% CI)
Preparation of food/ eating	Fetch water from pump	Lower and upper	58 (41.4%)	72 (51.4%)	46 (80.7%)	10 (17.5%)	2.9 (1.6–5.3)
	Preparing ugali*	Lower and upper	102 (72.9%)	28 (20%)	55 (96.5%)	0 (0%)	23.4 (1.5–377.7)
	Pour water from a bottle into a glass	Upper	129 (92.1%)	10 (7.1%)	57 (100%)	0 (0%)	8.6 (0.5–145.0)
	Cut vegetables with a knife	Upper	115 (82.1%)	14 (10%)	52 (91.2%)	2 (3.5%)	2.8 (0.7–12.1)
Clothing/personal care taking	Put on a T-shirt	Upper	129 (92.1%)	10 (7.1%)	56 (98.2%)	1 (1.8%)	4.1 (0.5–31.1)
	Wash yourself	Upper	124 (88.6%)	15 (10.7%)	56 (98.2%)	1 (1.8%)	6.1 (0.8–45.2)
	Clean yourself after going to the toilet	Upper	131 (93.6%)	8 (5.7%)	57 (100%)	0 (0%)	7.0 (0.4–119.2)
Working	Use a slasher*	Lower and upper	70 (50%)	53 (37.9%)	47 (82.5%)	7 (12.3%)	3.1 (1.5–6.4)
	Carrying loads on the head	Lower and upper	60 (42.9%)	46 (32.9%)	38 (66.7%)	11 (19.3%)	1.7 (1.0–3.0)
	Carrying harvest home	Lower and upper	68 (48.6%)	56 (40%)	44 (77.2%)	10 (17.5%)	2.3 (1.3–4.1)
	Open a bottle with screw top	Upper	124 (88.6%)	15 (10.7%)	57 (100%)	0 (0%)	12.8 (0.8–209.6)
	Tie a knot	Upper	123 (87.9%)	16 (11.4%)	56 (98.2%)	1 (1.8%)	6.5 (0.9–48.0)
Mobility	Walk level ground	Lower	116 (82.9%)	23 (16.4%)	55 (96.5%)	2 (3.5%)	4.7 (1.1–19.2)
	Walk uphill	Lower	67 (47.9%)	71 (50.7%)	46 (80.7%)	11 (19.3%)	2.6 (1.5–4.6)
	Walk downhill	Lower	76 (54.3%)	62 (44.3%)	50 (87.7%)	7 (12.3%)	3.6 (1.8–7.4)
	Run	Lower	58 (41.4%)	78 (55.7%)	46 (80.7%)	11 (19.3%)	2.9 (1.7–5.0)
	Squat	Lower	93 (66.4%)	46 (32.9%)	52 (91.2%)	5 (8.8%)	3.7 (1.6–8.9)
	Kneel	Lower	91 (65%)	48 (34.3%)	53 (93%)	4 (7%)	4.9 (1.8–12.9)
	Stand up from the floor	Lower and upper	90 (64.3%)	49 (35%)	50 (87.7%)	7 (12.3%)	2.8 (1.4–5.9)

*In the original BUFLS tool, “ugali” was listed as “fufu,” and “slasher” as “machete.”

*Note: Prevalence ratios for activities where zero events were recorded in the control group should be interpreted with caution; these wide confidence intervals reflect sparse data rather than precise effect sizes*

A floor effect was present in BUFLS scores, as 30.0% (n = 59) of respondents scored the lowest possible BUFLS score of 0. Among individuals in the SB/SBE group, 17.1% (n = 24) had a BUFLS score of 0, indicating no detectable functional limitation. The ceiling effect was not present, as none of the participants scored 100. The maximum BUFLS score was 86.8.

## Discussion

Our study results highlight that a history of snakebite envenoming is associated with a considerable burden of long-term disability among survivors in Kenya. WHODAS 2.0 scores revealed that roughly three out of four snakebite survivors had a persistent disability, compared with about one out of four individuals in the control group. Survivors who experienced more severe symptoms (SBE group) were confirmed to carry the highest burden. The snakebite survivors recorded significantly higher scores in all six WHODAS 2.0 domains relative to controls. The greatest impairments were observed in mobility, life activities, and participation, which reflect essential daily functions such as walking, farming, carrying loads, and fulfilling social roles. The prevalence of mild disabilities among controls reflects real-world background disability common in rural sub-Saharan African communities, which may arise from other prevalent physical or mental health conditions. The matching strategy deliberately did not account for background disability, as capturing this background level of functional difficulty was an important design choice. This allowed the estimation of the additional burden attributable to snakebite relative to the general level of functional limitation experienced in these communities, without the risk of overestimating the specific impact of snakebite.

Disability (as measured by WHODAS 2.0) and functional limitation (as measured by BUFLS) represent related but distinct constructs. The findings of this study provided compelling evidence that supports the validity of the BUFLS as a contextually appropriate tool for assessing functional limitations among snakebite survivors in Kenya. Originally developed for Buruli ulcer, a condition that, like cytotoxic snakebite envenoming, leads to tissue necrosis, contractures, and long-term physical impairment [29], the BUFLS demonstrated strong construct and discriminant validity in this new context with snakebites primarily leading to cytotoxicity. Even though the correlation between BUFLS and the WHODAS mobility domain (ρ = 0.87) exceeded our predefined range, this does not clearly indicate measurement redundancy as approximately 24% of variance remains unexplained. Additionally, the BUFLS is designed to capture limitations of the upper and lower extremities that manifest as restrictions in daily activities [28], which are not individually assessed by the WHODAS 2.0 mobility domain. Moreover, the BUFLS is likely to be more responsive in assessing interventions aimed at mitigating cytotoxic sequelae, as it measures localised limb function and activities rather than the broader domains covered by the WHODAS 2.0. The BUFLS is therefore a complementary instrument that identifies which specific tasks are impaired and can provide rehabilitation specialists with actionable targets.

A similar study conducted in Ghana [17], found that snakebite survivors (54%) experienced higher levels of disability than community controls (29%), but the overall pattern of impairment differed from our findings. In Ghana, visible sequelae were less common, and although WHODAS 2.0 captured disabilities after snakebite, BUFLS failed to distinguish survivors from controls. The different pattern likely reflects differences in species distribution and clinical presentation: *Echis ocellatus*, which predominates in northern Ghana, is strongly associated with systemic effects such as coagulopathy and haemorrhage but less frequently leads to local tissue damage and long-term functional disability [17]. By contrast, in our setting, cytotoxic snake species represent the predominant clinical problem, as evidenced by hospital reports, leading to local tissue damage, contractures, and amputations that BUFLS was well-suited to capture.

The long-term disability burden we identified among snakebite survivors in our study reflects patterns reported for other neglected tropical diseases (NTDs) across Africa and Asia. In Nigeria for example, persisting disabilities such as limb deformities and amputations were reported in over 11% of survivors identified through health facility records [30]. In Kenya, studies on NTDs such as schistosomiasis, trachoma and soil-transmitted helminths have shown that affected individuals face not only physical morbidity but also stigma, social exclusion and diminished wellbeing, impacts that often persist even when the clinical symptoms are mild or medically treatable [31,32]. Ochola et al. documented similar themes of permanent functional impairments, exclusion and the need for psychosocial support, underlining that disability in NTD contexts often transcends biomedical outcomes to encompass social and economic dimensions. These patterns are consistent with our WHODAS 2.0 findings, where survivors reported not only impairments in mobility and self-care but also in cognition, social interactions and participation, suggesting that snakebite, like other NTDs, has consequences that extend far beyond the acute phase of envenoming.

This study provides the first assessments of disabilities and functional limitations among snakebite survivors in Kenya. By employing both the WHODAS 2.0 and the BUFLS, we were able to generate a multidimensional understanding of post-snakebite disability, capturing both generic disabilities (WHODAS 2.0) and task-specific functional limitations (BUFLS), while validating the BUFLS as a tool to assess functional limitations among snakebite survivors.

A few limitations warrant consideration. Psychological impact of snakebite envenoming includes anxiety, depression and post-traumatic stress disorder [32,33]. WHODAS 2.0 includes items related to emotional wellbeing and social participation that may reflect some degree of psychological impact. However, it was not designed as a psychological assessment tool and may systematically underestimate the burden of anxiety, depression, and PTSD following snakebite. In addition, Community-based listing and voluntary participation may have resulted in individuals with more pronounced or visible sequelae being more likely to participate due to greater perceived need or interest, potentially leading to an overestimation of disability prevalence among all snakebite survivors in these communities. Classification of snakebite survivors into SB versus SBE was based exclusively on retrospectively reported clinical symptoms, and may be subject to recall bias and misclassification. Independent clinical verification was not possible owing to the study design and the limited availability of facility records, which typically lack sufficient detail on clinical symptoms to enable accurate classification. Although predefined clinical criteria were used to improve consistency, some non-differential misclassification may have occurred. Future studies would benefit from prospective clinical assessment and improved access to medical records to strengthen severity classification. Nonetheless, this approach helped to assess whether our instruments yielded higher scores among individuals with more severe symptom profiles, as anticipated. We also decided not to produce DALY estimates as snakebite survivors cannot provide data on mortality and the heterogeneity of clinical presentations after snakebites does not allow for extrapolation across countries or patient categories.

## Conclusion

A large proportion of snakebite survivors in Kenya experience substantial and enduring disability, with marked impairments in gross motor function, mobility, and social participation. These limitations significantly affect daily living, productivity, and reintegration, highlighting the need to recognize snakebite not only as an acute medical emergency but also as a chronic disabling condition. Based on our findings, the applicability of BUFLS is probably limited to regions where cytotoxic envenoming predominates.

These findings offer direct implications for improving care after SBE. Snakebite in rural Kenya is associated with persistent functional impairment that highlights the need for structured follow-up mechanisms, rehabilitation programmes and comprehensive psychosocial support. Strengthening care coordination between community health units and higher-level facilities could improve continuity of care beyond hospital discharge, the evaluation of which requires a combination of validated tools that both align with targeted rehabilitation goals and are sufficiently responsive in evaluating interventions.

## Supporting information

S1 Table Activities in the BUFLS reported as not being performed among snakebite survivors and controls. (PDF)
