# Supplementary material for: Evaluation of long-term consequences among snakebite survivors in rural Kenya including validation of a functional limitations assessment tool
Source: PLoS Negl Trop Dis. 2026 Apr 28;20(4):e0014259. doi: 10.1371/journal.pntd.0014259 (PMC13152180; doi:10.1371/journal.pntd.0014259)
Supplement: S1 Table — (PDF) [file pntd.0014259.s001.pdf]

Supplementary Table S1. Activities in the BUFLS reported as not being performed among snakebite survivors and controls.

Type of activity	Activity	Extremity involved	Snakebite survivors (n = 140)	Controls (n = 57)
Preparation of food / eating	Fetch water from pump	Lower and upper	10 (7.1%)	1 (1.8%)
	Preparing ugali*	Lower and upper	10 (7.1%)	2 (3.5%)
	Pour water from a bottle into a glass	Upper	1 (0.7%)	0 (0%)
	Cut vegetables with a knife	Upper	11 (7.9%)	3 (5.3%)
Clothing / personal care taking	Put on a T-shirt	Upper	1 (0.7%)	0 (0%)
	Wash yourself	Upper	1 (0.7%)	0 (0%)
	Clean yourself after going to the toilet	Upper	1 (0.7%)	0 (0%)
Working	Use a slasher*	Lower and upper	17 (12.1%)	3 (5.3%)
	Carry loads on the head	Lower and upper	34 (24.3%)	8 (14.0%)
	Carry harvest home	Lower and upper	16 (11.4%)	3 (5.3%)
	Open a bottle with screw top	Upper	1 (0.7%)	0 (0%)
	Tie a knot	Upper	1 (0.7%)	0 (0%)
Mobility	Walk level ground	Lower	1 (0.7%)	0 (0%)
	Walk uphill	Lower	2 (1.4%)	0 (0%)
	Walk downhill	Lower	2 (1.4%)	0 (0%)
	Run	Lower	4 (2.9%)	0 (0%)
	Squat	Lower	1 (0.7%)	0 (0%)
	Kneel	Lower	1 (0.7%)	0 (0%)
	Stand up from the floor	Lower and upper	1 (0.7%)	0 (0%)
